# RETHINKING THE PLACE OF PLACE FOR AGING: ENDURING AND NEW CHALLENGES FOR ENVIRONMENTAL GERONTOLOGY

**DOI:** 10.1093/geroni/igae098.1281

**Published:** 2024-12-31

**Authors:** Frank Oswald

**Affiliations:** Goethe University Frankfurt, Frankfurt am Main, Hessen, Germany

## Abstract

Environmental gerontology emphasizes the relationship between place and aging from a conceptual perspective while also aiming to empirically analyze person-environment (P-E) relationships to improve quality of life and health in later life. After decades of empirical research, a critical discussion of theorizing place and aging is needed to better address not only enduring but also new challenges of P-E processes in rapidly changing societies worldwide, including challenges of housing, mobility, accessibility and participation for older adults in the “third” and “fourth” age. This contribution aims to (1) rethink the place of place for aging in modern societies from different disciplines, (2) identify a set of enduring and novel issues of P-E relationships. This is underpinned by specific references to empirical findings from selected studies, often over-representing ageing in Western societies. An example of an enduring issue would be the essential understanding of how an aging individual interacts with the home environment. An example of a novel issue would be technology use in later life in an increasingly digitalized world. Moreover, the contribution aims to (3) provide an overview over selected theories addressing place and aging interrelations, and emphasizing fundamental concepts such as interaction, transaction, co-construction and co-constitution to understand P-E interrelations, and finally to (4) offer a synthesis of how theories can address enduring and novel challenges and identify open questions that remain.

